# Coumestrol Epigenetically Suppresses Cancer Cell Proliferation: Coumestrol Is a Natural Haspin Kinase Inhibitor

**DOI:** 10.3390/ijms18102228

**Published:** 2017-10-24

**Authors:** Jong-Eun Kim, Sung-Young Lee, Mi Jang, Hyo-Kyung Choi, Jong Hun Kim, Hanyong Chen, Tae-Gyu Lim, Zigang Dong, Ki Won Lee

**Affiliations:** 1 Research Institute of Biotechnology and Medical Converged Science, Dongguk University-Seoul, Goyang 10326, Korea; jekim14@dongguk.edu; 2 The Hormel Institute, University of Minnesota, Minneapolis, MN 55912, USA; mbiotec@hotmail.com (S.-Y.L.); hchen@hi.umn.edu (H.C.); 3 Korea Food Research Institute, Iseo-myeon, Wanju-gun, Jeollabuk-do 55365, Korea; jangmi@kfri.re.kr (M.J.); chkyoung@kfri.re.kr (H.-K.C.); 4 Research Institute of Agriculture and Life Sciences, Seoul National University, Seoul 08826, Korea; rose-0@snu.ac.kr; 5 Department of Agricultural Biotechnology, Seoul National University, Seoul 08826, Korea

**Keywords:** cancer, coumestrol, haspin kinase, histone H3

## Abstract

Targeting epigenetic changes in gene expression in cancer cells may offer new strategies for the development of selective cancer therapies. In the present study, we investigated coumestrol, a natural compound exhibiting broad anti-cancer effects against skin melanoma, lung cancer and colon cancer cell growth. Haspin kinase was identified as a direct target protein of coumestrol using kinase profiling analysis. Histone H3 is a direct substrate of haspin kinase. We observed haspin kinase overexpression as well as greater phosphorylation of histone H3 at threonine 3 (Thr-3) in the cancer cells compared to normal cells. Computer modeling using the Schrödinger Suite program identified the binding interface within the ATP binding site. These findings suggest that the anti-cancer effect of coumestrol is due to the direct targeting of haspin kinase. Coumestrol has considerable potential for further development as a novel anti-cancer agent.

## 1. Introduction

Cancer is the leading cause of death worldwide, with approximately 14 million new cases and 8.2 million cancer related deaths in 2012 [1]. In 2015, 1,658,370 new cancer cases and 589,430 cancer deaths were reported in the United States [2]. The representative feature of cancer is aberrant growth of tissue by alterations of oncogenes and/or tumor suppressors [3]. From this mutation of oncogenes, various changes occur, such as transcription factor alteration, chromatin remodeling and inducing/reducing signaling pathways [3]. Epigenic changes are particularly widely detected in several cancer cases [4]. In the previous literature, 147 and 27 genes are hypermethylated and hypomethylated, respectively.

Haspin kinase is a serine/threonine kinase and is conserved in many eukaryotic lineages including animals, fungi, and plants. During mitosis, haspin kinase directly phosphorylates histone H3 at the Thr-3 site [5]. Haspin kinase is expressed in various tissues, such as testis, bone marrow and thymus. In particular, highly expressed haspin kinase was shown in proliferating cells [6]. Recently, haspin kinase has been the focus of anti-cancer drugs [7,8]. Indeed, the siRNA of haspin represented the premature loss of centromeric cohesion in mitosis, and consequently mitosis was arrested [9]; or, vice versa, overexpressed haspin kinase interrupted the normal dissociation of cohesion [10]. In cancer cases, anti-haspin kinase agents have been discovered as anti-tumor agents [7,8]. Huertas D. et al. reported an anti-tumor activity of haspin kinase inhibitor (CHR-6494) using mouse xenograft model [11]. Overall, inhibition of haspin kinase activity can be regarded as a promising strategy for general cancer drugs.

Coumestrol is one of the chemicals in the coumestan family and presents in soybean legumes, brussels sprouts, and spinach [12]. Interestingly, after stimulation such as germination and chemical elicitors, daidzein, one of the isoflavones, is converted to coumestrol [13]. A previous paper has indicated the estrogenic activity of coumestrol [14]. Furthermore, casein kinase 2 (CK2) was recently suggested as the target molecule of coumestrol [15,16,17]. In particular, these previous studies represent anti-breast cancer [15] and prostate cancer [16] activities by direct CK2 suppression. However, the epigenetic regulatory activity of coumestrol remains unclear in anti-cancer effects.

In this study, we sought the general anti-cancer effect of coumestrol. To unveil the molecular target of coumestrol, we referenced our previous published paper [18]. Haspin kinase was suggested as the target protein of coumestrol based on the screening analysis of 259 kinases, and we found that the inhibition of hapin kinase activity by coumestrol resulted in the suppression of cancer cell growth.

## 2. Results

### 2.1. Coumestrol Inhibits the Growth of Various Cancer Cell Types

**Figure 1:**
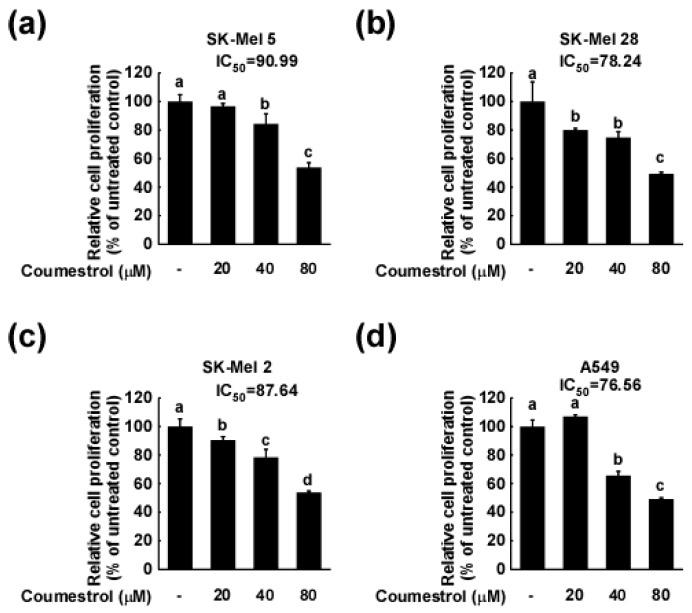

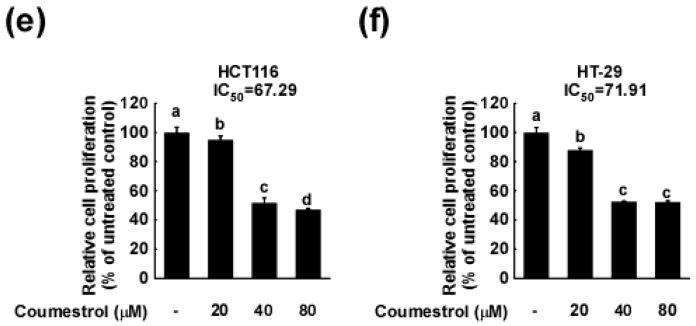
Anti-cancer cell growth effect of coumestrol in several cancer cell lines, (**a**) SK-Mel5; (**b**) SK-Mel 28; (**c**) SK-Mel 2; (**d**) A549; (**e**) HCT116; and (**f**) HT-29. 2 × 10^3^ cells were seeded to each well of a 96-well plate, before 20, 40 or 80 μM of coumestrol was added as treatment. Cell growth was measured using Cell Titer96 Aqueous One Solution (Promega) as described in the Materials and Methods. Each data is the mean ± standard deviation of three replicates. Different letters indicate the significant differences at *p* < 0.05 by Turkey multiple range tests.

Uncontrolled growth is a hallmark property of cancer cells [19], and the suppression of such growth has served as the basis for developing numerous anti-cancer agents [20,21]. We investigated the anti-growth effect of coumestrol on various cancer cells. As indicated in Figure 1a–c, we observed a dose-dependent inhibition of cancer cell growth following coumestrol treatment in skin melanoma cells (SK-Mel 5 (Figure 1a), SK-Mel 28 (Figure 1b), and SK-Mel 2 (Figure 1c)). Furthermore, the anti-cancer activity of coumestrol was also observed in lung cancer cells (A549, Figure 1d) as well as colon cancer cells (HCT 116 (Figure 1e) and HT-29 (Figure 1f)). The dose range of coumestrol used in these assays did not cause observable cytotoxicity in HCT116 and SK-Mel 5.

### 2.2. Coumestrol Reduces Anchorage-Independent Cancer Cell Growth

**Figure 2:**
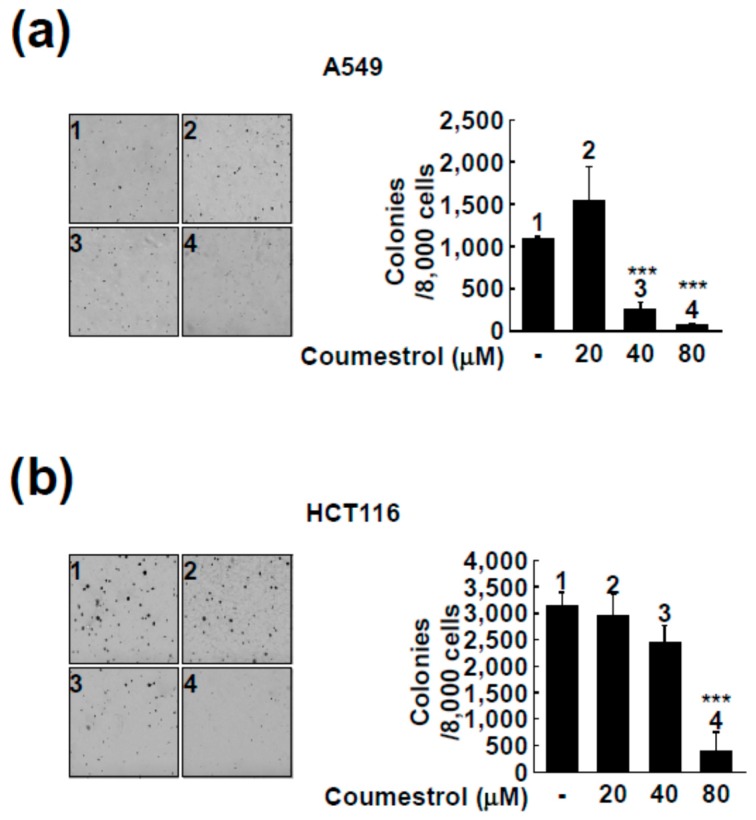

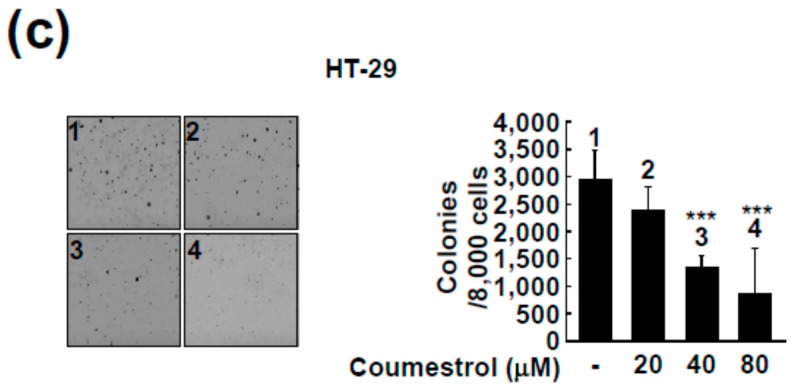
Inhibitory effect of coumestrol on anchorage-independent cell growth of lung cancer cells (**a**) and colon cancer cells (**b**,**c**). The detailed procedure is described in the Materials and Methods. Briefly, the colony formation of the cancer cells was tested with 0.3% agar matrix as the top layer with 0.6% agar matrix as the base layer. The colony formation was captured with the X 40 magnification. Data are representative of three independent experiments, which gave similar results. The asterisks (***) indicate a significant difference of *p* < 0.001 compared to the untreated group.

Contrary to normal cells, cancer cells can grow in 0.3% agar conditions by colony formation [22]. We next investigated the effect of coumestrol on anchorage-independent growth in three different cancer cell lines (A549 (Figure 2a), HCT116 (Figure 2b), and HT-29 (Figure 2c)). Consistent with the findings in Figure 1, coumestrol significantly attenuated colony formation by cancer cells (Figure 2). As seen in Figure 2, 80 μM of coumestrol caused an over 50% reduction in colony formation.

### 2.3. Haspin Kinase Is a Direct Target of Coumestrol

**Figure 3:**
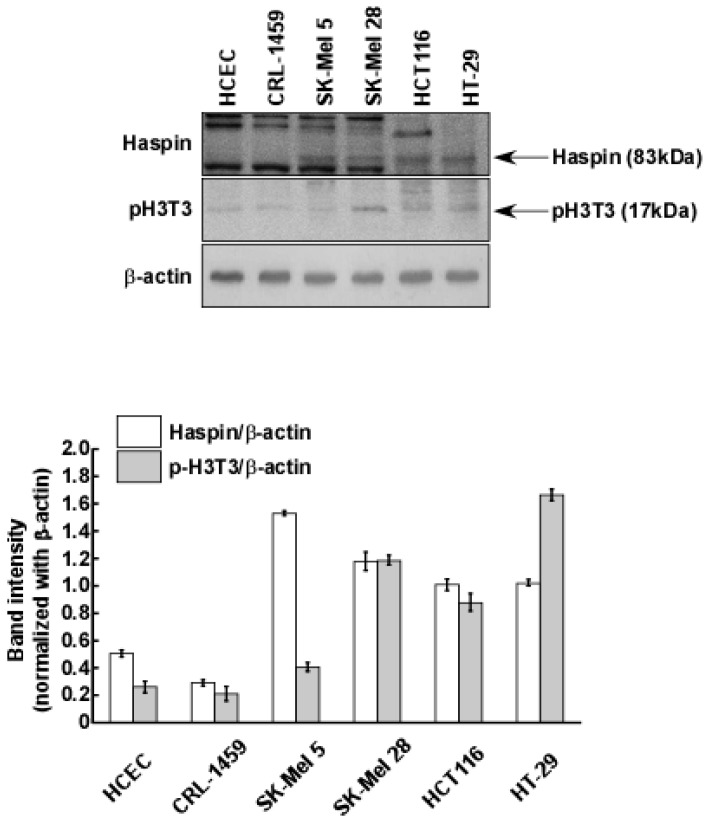
Haspin kinase is overexpressed and histone H3 is phosphorylated in cancer cells compared to normal cells. Protein levels were detected with the specific primary antibodies. Data are representative of three independent experiments, which gave similar results.

**Figure 4:**
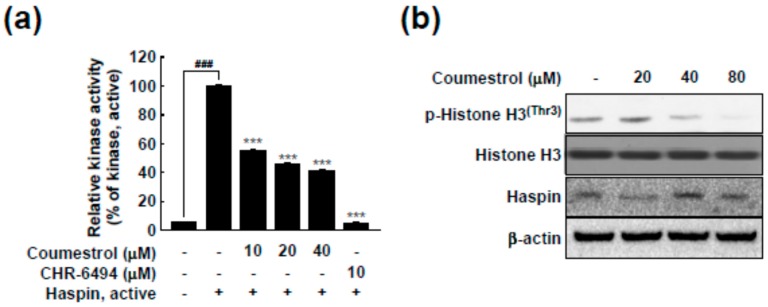
Coumestrol directly suppresses haspin kinase activity and the phosphorylation of histone H3 at Thr-3. (**a**) The effect of coumestrol on haspin kinase activity was investigated using recombinant haspin kinase. CHR-6494, a haspin kinase inhibitor, was used as a positive control; and (**b**) The effect of coumestrol on the haspin downstream signaling pathway was confirmed. Protein levels were detected with specific primary antibodies. Data are representative of three independent experiments, which gave similar results. The pound (^###^) signs indicate a significant difference at *p* < 0.001, compared to the untreated control and the asterisks (***) indicate a significant difference at *p* < 0.001 compared to the kinase treated group.

We previously screened for target proteins of coumestrol using kinase profiling analysis (KinaseProfilerTM service (MERCK Millipore)) [18]. Based on the previous results, haspin kinase was identified as the most suppressed target kinase in the presence of 5 μM of coumestrol [18]. Because haspin kinase directly phosphorylates histone H3 at the Thr-3 site during mitosis [5,23], it has been regarded as a functional target for anti-cancer drugs [7,8]. Indeed, we verified a high level of expression of haspin kinase and the phosphorylation of histone H3 at the Thr-3 site in the cancer cells (SK-Mel 5, SK-Mel 28, HCT116 and HT-29) compared to normal cells (HCEC; human colonic epithelial cells and CRL-1459) (Figure 3). Thus, we confirmed the inhibitory effect of coumestrol on haspin kinase activity using recombinant haspin kinase. In Figure 4a, coumestrol dose-dependently reduced haspin kinase activity. In this assay, CHR-6494, a haspin kinase inhibitor, was used as a positive control. Haspin kinase was discovered to be an upstream regulator of histone H3 by phosphorylation at Thr-3 of histone H3 [5,23]. To evaluate whether the effect of coumestrol on haspin kinase activity occurs in the cells, the phosphorylation level of histone H3 was examined using a specific antibody against histone H3 phosphorylation at the Thr-3 site after coumestrol treatment. As shown in Figure 4b, the phosphorylation level of histone H3 was reduced by coumestrol treatment in the HCT116 cells.

### 2.4. Coumestrol Directly Interacts with Haspin Kinase

**Figure 5:**
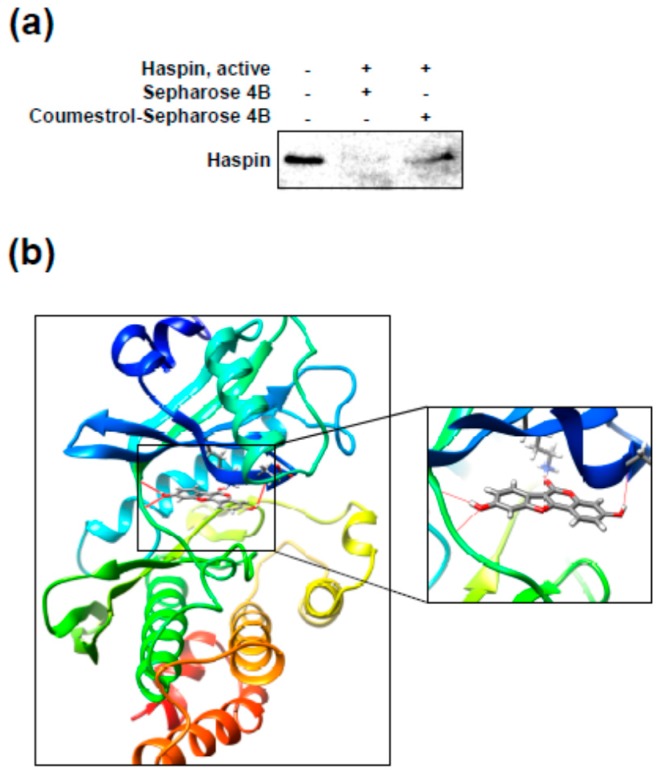
Coumestrol directly binds with haspin kinase. (**a**) Evidence for a direct interaction between coumestrol and recombinant haspin kinase. The binding was confirmed with a pull-down assay as described in the Materials and Methods. Data are representative of three independent experiments, which gave similar results; and (**b**) Modeling of coumestrol with haspin kinase. The modeling results were obtained using Schrödinger Suite 2015.

We next investigated the direct interaction between coumestrol and haspin kinase using pull-down assay with Sepharose 4B beads. As shown in Figure 5a, the haspin protein was detected in coumestrol–Sepharose 4B lane (in the third lane). However, coumestrol was not pulled down by Sepharose 4B alone (in the second lane). To better understand how coumestrol interacts with haspin, we used a computational docking model with the Glide docking program in Schrödinger Suite 2015. In the docked models, coumestrol can theoretically dock at the ATP binding pocket of haspin. Some important structural hydrogen bonds are likely formed between coumestrol and haspin (Figure 5b) (some images were generated with the UCSF; University of California, San Francisco Chimera program [24]). The pull-down assay further confirmed this direct interaction between coumestrol and haspin kinase.

### 2.5. Haspin Kinase Modulates Cancer Cell Growth but Not Casein Kinase 2

**Figure 6:**
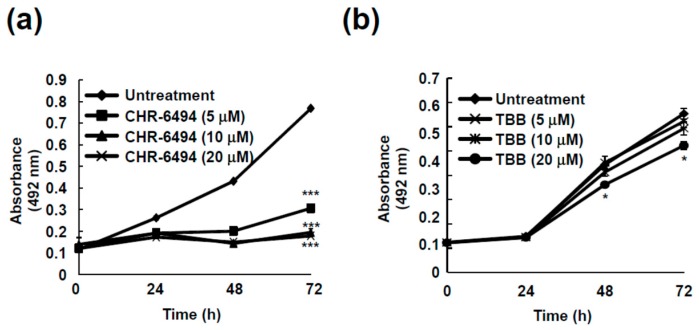
Cancer cell growth is modulated by haspin kinase but not casein kinase 2. (**a**) CHR-6494, a haspin kinase inhibitor and (**b**) TBB, a casein kinase 2 inhibitor, were treated to the cells and HCT116 cell growth was estimated using Cell Titer96 Aqueous One Solution (Promega) as described in the Materials and Methods. The asterisk (* and ***) indicates a significant difference (of *p* < 0.01 and *p* < 0.001, respectively) compared to the untreated group.

To understand the relevance of inhibiting haspin kinase and its effect on cancer cell growth, we measured the cell growth in HCT116 cells after treatment with CHR-6494, a commercial haspin kinase inhibitor. HCT116 cell growth was dramatically reduced by CHR-6494 treatment (Figure 6a). Even 5 μM of CHR-6494 resulted in more than 50% suppression of cancer cell growth (Figure 6a).

Based on recent findings, coumestrol has been proposed as a CK2 inhibitor [15,17]. To investigate whether the anti-cancer effect of coumestrol is the result of CK2 inhibition, we tested the effect of TBB (4,5,6,7-tetrabromobenzotriazole), a commercial CK2 inhibitor, on HCT116 cell growth. As seen in Figure 6b, TBB did not show significant activity toward cancer cell growth over 72 h. Although CK2 is a known target protein of coumestrol [15,17], CK2 does not regulate cancer cell growth.

## 3. Discussion

Many anti-cancer drugs have failed in clinical development due to unacceptable toxicity and side effects. Thus, numerous efforts have been made to develop general anti-cancer drugs with improved safety profiles. Normal cells are transformed into carcinogenic cells via the alterations of oncogenes and/or tumor suppressors during the process of carcinogenesis. In such a step, several genes are often epigenetically modified, such as by chromatin remodeling [3]. Haspin kinase modulates mitosis in cells by directly phosphorylating histone H3 at Thr-3 [5]. Because haspin kinase regulates the proliferation of cells, haspin kinase inhibitors have been developed as anti-cancer drugs [7,8]. Treatment with CHR-6494, a haspin kinase inhibitor, reduces angiogenesis and tumor growth in a mouse model with no evidence of toxicity [11].

We previously reported kinase profiling results for coumestrol [18], and Flt3 was selected as a target protein of coumestrol. However, as well as Flt3, the activity of numerous kinases was significantly reduced by coumestrol treatment [18]. Indeed, we observed a dose-dependent reducing effect of coumestrol on recombinant haspin kinase activity. Furthermore, reduced histone H3 phosphorylation was verified by coumestrol treatment in HCT116 cells. The computer docking model results implicated that coumestrol can theoretically binds to ATP binding pocket of haspin in Figure 5b. Haspin kinase is involved in cell proliferation [6], and the inhibition of cancer cell proliferation resulted from the suppression of haspin kinase activity by coumestrol. Therefore, we postulated that haspin is another functional target of coumestrol for suppressing cancer cell proliferation. Additionally, anchorage-independent colony formation was attenuated by coumestrol treatment. Previous studies have reported that CK2 is a target protein of coumestrol [15,16,21]. Indeed, our previous kinase profiling results indicated that the activity of CK2 is dramatically reduced by coumestrol [18]. Thus, we sought to confirm whether CK2 is involved in cancer cell growth in HCT116 cells using TBB, a pharmaceutical inhibitor of CK2. Although CHR-6494 caused a significant suppressive effect on cancer cell growth, TBB did not show a significant effect. This result is notable in that, although CK2 is a target protein of coumestrol, it is not a major protein responsible for cancer cell growth. Consequently, coumestrol-inhibited cancer cell proliferation is a result of the suppression of haspin kinase activity but not CK2.

Although a clinical trial and in vivo studies are required to confirm the anti-cancer effects of coumestrol, our findings confirm that coumestrol possesses anti-cancer properties and modulates haspin kinase.

## 4. Materials and Methods

### 4.1. Reagents

Coumestrol and anti-β-actin were obtained from Sigma-Aldrich. McCoy’s 5A, DMEM and RPMI 1640 medium were purchased from Thermo Fisher Scientific (Carlsbad, CA, USA)). Basal Medium Eagle (BME), gentamicin, penicillin/streptomycin, and L-glutamine were purchased from Invitrogen (Carlsbad, CA, USA). [γ-32P]-ATP and the chemiluminescence detection kit were obtained from Amersham Pharmacia Biotech (Piscataway, NJ, USA). CNBr-Sepharose 4B beads were purchased from GE Healthcare (Pittsburgh, PA, USA). The MTS solution was purchased from Promega (Madison, WI, USA). Antibodies against phospho-histone H3 (Thr-3), histone H3 and haspin were obtained from Cell Signaling Technology (Danvers, MA, USA).

### 4.2. Cell Culture

All cell lines were obtained from the American Type Culture Collection (Manassas, VA USA). HCT116 and HT-29 cells were culture in McCoY’s 5A medium supplemented with 10% (*v/v*) FBS (Atlanta Biologicals). SK-Mel 5, SK-Mel 28 and SK-Mel 2 cells were grown in MEM containing 10% (*v/v*) FBS. A549 cells were cultured in F-12K medium supplemented with 10% (*v/v*) FBS. Each group of cells were transferred to new plate when the cells confluency reached at around 90%.

### 4.3. Cell Proliferation Assay

The cells were seeded (2 × 10^3^ cells/well) in 96-well plates and incubated for 12 h. Coumestrol was then treated at various concentrations for 72 h. The Cell Titer96 Aqueous One Solution (Promega) was added to the wells (20 μL/well) and incubated for 1 h at 37 °C in 5% CO_2_. Absorbance was analyzed at 492 nm.

### 4.4. Anchorage-Independent Cell Growth

8 × 10^3^ cells were suspended in BME supplemented with 10% FBS, 1% antibiotics and 0.3% agar with different doses of coumestrol in a top layer. The base layer was composed with 0.6% agar with different dose of coumestrol. The plates were incubated at 37 °C in 5% CO_2_ for 2 weeks, before colony numbers were counted under a microscope using the Image-Pro Plus Software version 4 program (Media Cybernetics).

### 4.5. Preparation of Coumestrol-Sepharose 4B Beads

Sepharose 4B powder (0.3 g) was activated with 1 mmol/L HCl and coumestrol was conjugated to the activated Sepharose 4B beads in coupling solution (0.1 mol/L NaHCO_3_, pH 8.3 and 0.5 mol/L NaCl) by rotation overnight under 4 °C. Above coupling solution was used to wash out the mixture. And Sepharose 4B-fused coumestrol mixture was transferred to 0.1 mol/L Tris-HCl buffer (pH 8.3). The uncoupled-coumestrol was washed out using 0.1 mol/L acetate buffer (pH 4.0) and 0.1 mol/L Tris-HCl buffer (pH 8.0) containing 0.5 mol/L NaCl.

### 4.6. Pull down Assay

Recombinant protein (20 ng) or cell lysate (300 μg) from HCT116 cells were mixed with Sepharose 4B beads (as a negative control) or coumestrol-fused Sepharose 4B beads (100 μL) in reaction buffer [50 mmol/L Tris, pH 7.5, 150 mmol/L NaCl, 1 mmol/L dithiothreitol (DTT), 0.01% Nonidet P-40, 2 mg/mL BSA, 0.2 mmol/L phenylmethylsulfonylfluoride (PMSF), 5 mmol/L EDTA, and 1× protease inhibitor (PI) mixture]. The mixture was incubated by rotation overnight under 4 °C and washed with washing buffer (50 mmol/L Tris, pH 7.5, 0.01% Nonidet P-40, 5 mmol/L EDTA, 150 mmol/L NaCl, 1 mmol/L DTT, and 0.02 mmol/L PMSF). The binding was detected by using specific antibodies.

### 4.7. Kinase Assay

The haspin kinase assay was carried out using active recombinant haspin kinase (Millipore, Bedford, MA, USA) and the capacity of each kinase to transfer the radiolabeled phosphate from [γ-32P] ATP was measured according to the manufacturer’s instructions. In brief, active haspin kinase was co-incubated with each compound at 30 °C for 15 min. As a substrate, histone H3 (New England Biolabs) was added to each vial and then incubated at 30 °C for 15 min with [γ-32P] ATP solution in a magnesium acetate-ATP cocktail buffer (Upstate Biotechnology Inc., Lake Placid, NY, USA). The mixtures were then transferred onto p81 paper. Unreacted chemicals were washed out with 0.75% phosphoric acid, before the radiolabeled phosphate was measured using a scintillation counter.

### 4.8. Western Blot Analysis

The cells were cultured for 48 h (80% of confluency), before being treated in the presence or absence of coumestrol for 12 h. The protein was isolated from the cells by using lysis buffer 10 mmol/L Tris (pH 7.5), 150 mmol/L NaCl, 1% Triton X-100, 1 mmol/L DTT, 0.1 mmol/L PMSF, 5 mmol/L EDTA, and 10% glycerol and PI cocktail tablet and electrophoretically separated using SDS-PAGE. The separated proteins were moved on Immobilon-P membranes (Millipore Corporation). The membranes were blocked with 5% skim milk for 2 h and the specific primary antibodies were treated overnight. After hybridization with an HRP-conjugated secondary antibody, the bands were visualized using a chemiluminescence detection kit (GE Healthcare).

### 4.9. Molecular Modeling

Computer modeling of coumestrol binding with haspin was performed with the Schrödinger Suite 2015 program [25]. First, an X-RAY diffraction structure of human haspin in complex with AMP with a resolution of 1.85 Å (PDB ID 3DLZ) [26] was obtained from the RCSB Protein Data Bank [27]. This structure was prepared using the standard procedure in the Protein Preparation Wizard in Schrödinger Suite 2015. Hydrogen atoms were added consistent with a pH of 7 and all water molecules were removed. Finally, the ATP binding site based on a receptor grid was generated for the docking study.

Coumestrol was prepared using LigPrep in Schrödinger for docking by default parameters. Then coumestrol-haspin docking was accomplished using the program Glide with default parameters under extra precision (XP) to achieve the best-fitting representative structure.

### 4.10. Statistical Analysis

All experiments were performed at least three times. Statistical analysis was performed using one-way analysis of variance (ANOVA) followed by Turkey post hoc test for multiple comparison. The *p* value of less than 0.05 was considered as a significant difference. Statistical analysis was conducted using IBM SPSS statistics software (version 20, SPSS Inc., Chicago, IL, USA).
